# CT chest findings in patients infected with COVID-19: review of literature

**DOI:** 10.1186/s43055-020-00355-3

**Published:** 2020-11-27

**Authors:** Mohamed Mohamed Hefeda

**Affiliations:** Radiology Department, Tanta University, Tanta, Egypt

**Keywords:** Chest CT, Coronavirus, COVID-19 pneumonia, Pneumonia, Viral pneumonia

## Abstract

**Background:**

Coronavirus disease 2019 (COVID-19) is a highly infectious disease causing severe respiratory distress syndrome that was first discovered by the end of 2019 in Wuhan, China.

**Main text:**

A wide variety of CT findings in COVID-19 have been reported in different studies, and the CT findings differ according to the stage of the disease and disease severity and associated co-morbidities. We will discuss each sign separately and its importance in diagnosis and prognosis.

**Conclusion:**

CT plays a pivotal role in the diagnosis and management of COVID-19 pneumonia. The typical appearance of COVID-19 pneumonia is bilateral patchy areas of ground glass infiltration, more in the lower lobes. The appearance of other signs like consolidation, air bronchogram, crazy pavement appearance, and air bubble signs appear during the course of the disease. In the context of pandemic, the CT chest can be used as a screening tool in symptomatic patients as it is cheaper, available, and time saving.

## Background

Coronavirus, named after their crown-like appearance, is a large family of viruses that was first discovered by researchers in Chicago in 1965 [1]. The virus was not investigated again till 2003, when the severe acute respiratory distress syndrome (SARS) outbreak started in China and rapidly spread to 29 countries. The SARS outbreak infected a total of 8089 patients with high mortality rate (774 deaths in 17 countries). Ten years later, Middle East respiratory distress syndrome (MERS) outbreak started in Saudi Arabia, infected totally 2506, with total deaths of 862 [2, 3].

Coronavirus disease 2019 (COVID-19) is a highly infectious disease causing severe respiratory distress syndrome that was first discovered by the end of 2019 in Wuhan, China, and spread globally. On 11 March 2020, the pandemic of coronavirus disease 2019 has been declared by the World Health Organization as an international public health emergency. More than 5.3 million cases and 342,000 deaths were reported all over the world by 24 May 2020, and the disease was reported in 188 countries [4–6]. In literature, the mortality rate ranges from 4.3 to 15% [7–9]. The updated mortality rate is about 5.8% according to the online Johns Hopkins Center dashboard (retrieved 24 May 2020) [6]. The disease is transmitted by person to person (direct contact by exposure to expired air from infected person) and touching a surface contaminated from infected person (indirect contact) [10]. The disease affects males more than females probably due to immune-linked chromosomes or occupational exposure [11]. The incubation period is 2–14 days, average period 5.2 days [11]. The clinical picture ranges from simple lung infection to severe respiratory distress syndrome, metabolic acidosis, diarrhea, intestinal symptoms, coagulation dysfunction, and septic shock [11].

Rapid detection of COVID-19 is vital for early treatment of patients and more importantly for quick isolation of the patient to stop the chain of contamination.

Nowadays, the standard technique for confirming COVID-19 is the real-time polymerase chain reaction (RT-PCR). Other laboratory findings include low white blood cells, lymphopenia, thrombocytopenia, high serum C reactive protein, and elevated serum ferritin [12, 13].

Radiologic imaging, especially thin slice CT, has important roles in the diagnosis, management, and follow-up of patients with COVID-19 pneumonia. Chest CT can detect early phases of infection and enable early isolation of patients [14, 15].

The current review will discuss the main findings of COVID pneumonia in the literature, main differential diagnosis, and the relation between the CT appearance and the clinical severity and prognosis.

## CT signs of coronavirus pneumonia

The cell receptor of COVID-19 is angiotensin-converting enzyme-2 (ACV2) [16]. COVID-19 starts as interstitial pneumonitis and then affects lung parenchyma. A wide variety of CT findings in COVID-19 have been reported in the different studies, and the CT findings differ according to the stage of the disease and disease severity and associated co-morbidities. The current study will discuss each sign separately and its importance in diagnosis and prognosis.

### Ground glass opacity

Ground glass opacity (GGO) is the non-specific hazy opacification of the lung in the X-ray or computed tomography with no obliteration of bronchial or vascular markings. The presumed pathology include partial filling of the lung alveoli by fluid, interstitial thickening, or partial collapse of lung alveoli [17].

**Fig. 1:**
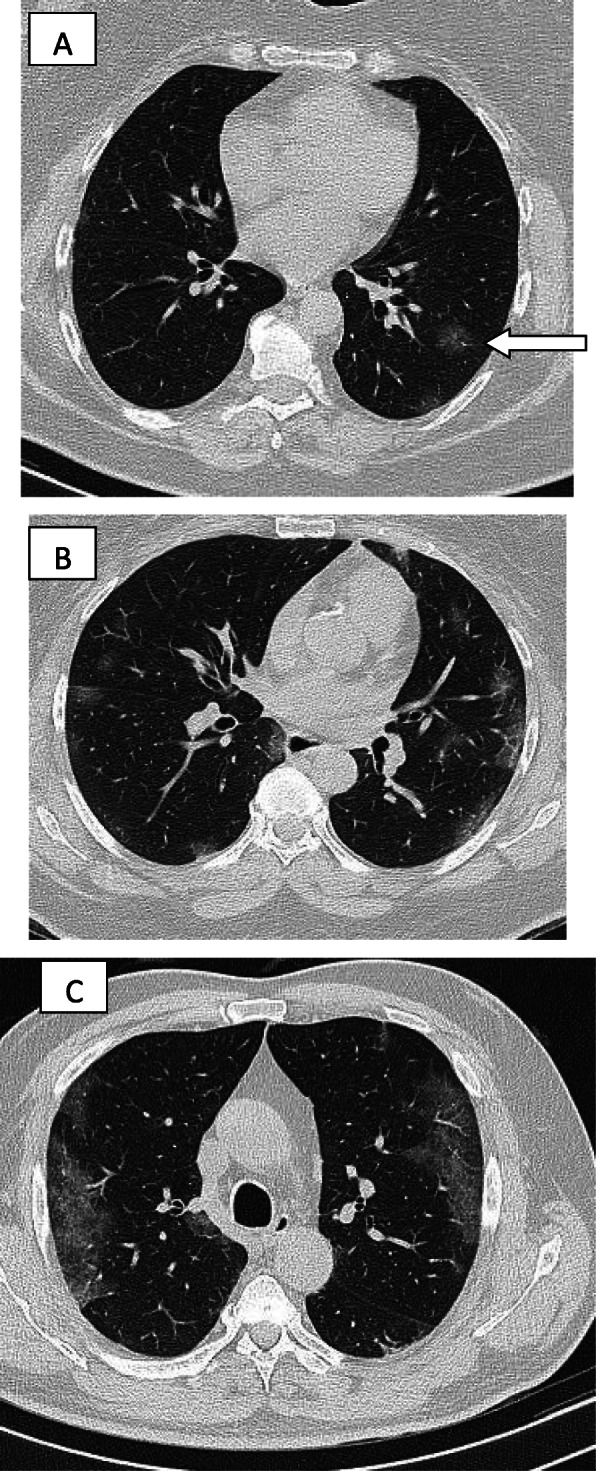
Three different cases of early COVID-19 pneumonitis. **a** Patient in second day after appearance of symptoms with ill-defined early ground glass infiltration patches in the left lower lobe. **b** Patient in third day of symptoms with multiple patchy areas of sub-pleural ground glass infiltration in both lungs. **c** Another patient 4 days after symptoms with bilateral wide areas of ground glass infiltration

**Fig. 2:**
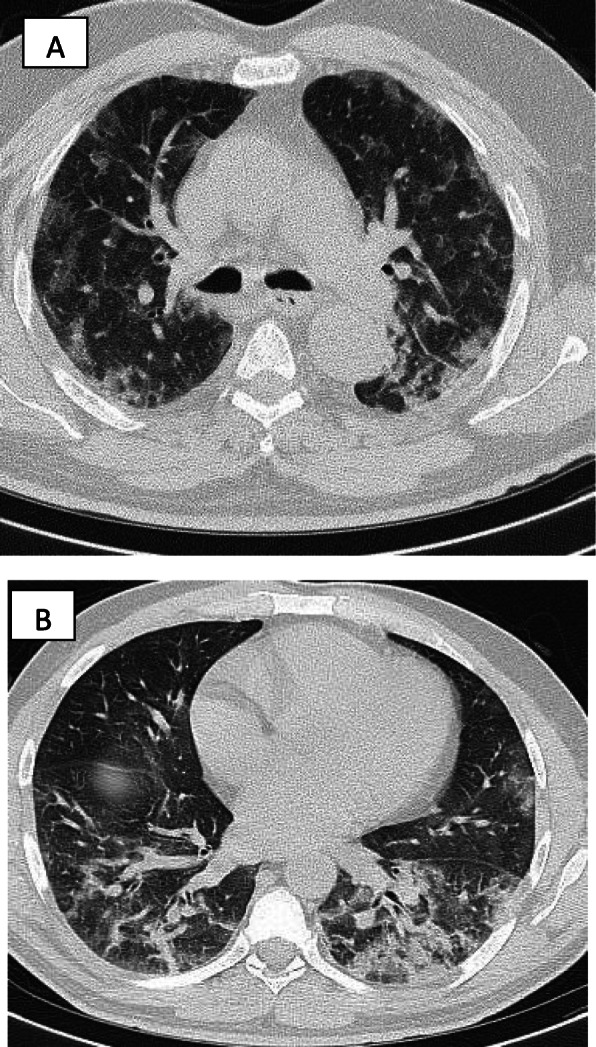
Two different cases with bilateral multiple patches of ground glass infiltration and subsegmental consolidation, lesions mainly peripheral and posterior

**Fig. 3:**
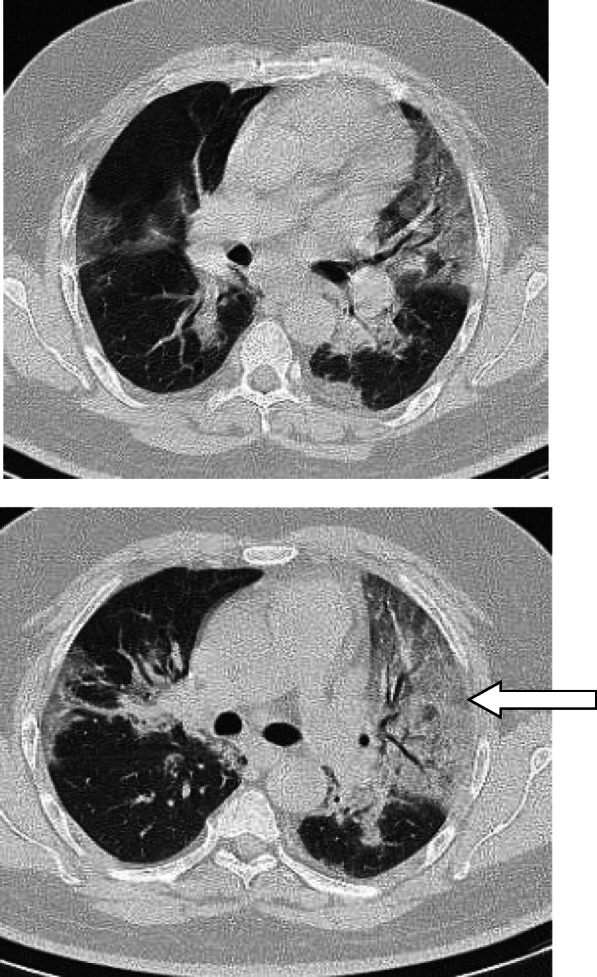
Wide areas of ground glass infiltration with air bronchogram. Patient with COVID-19 pneumonia 8 days after onset of symptoms

**Fig. 4:**
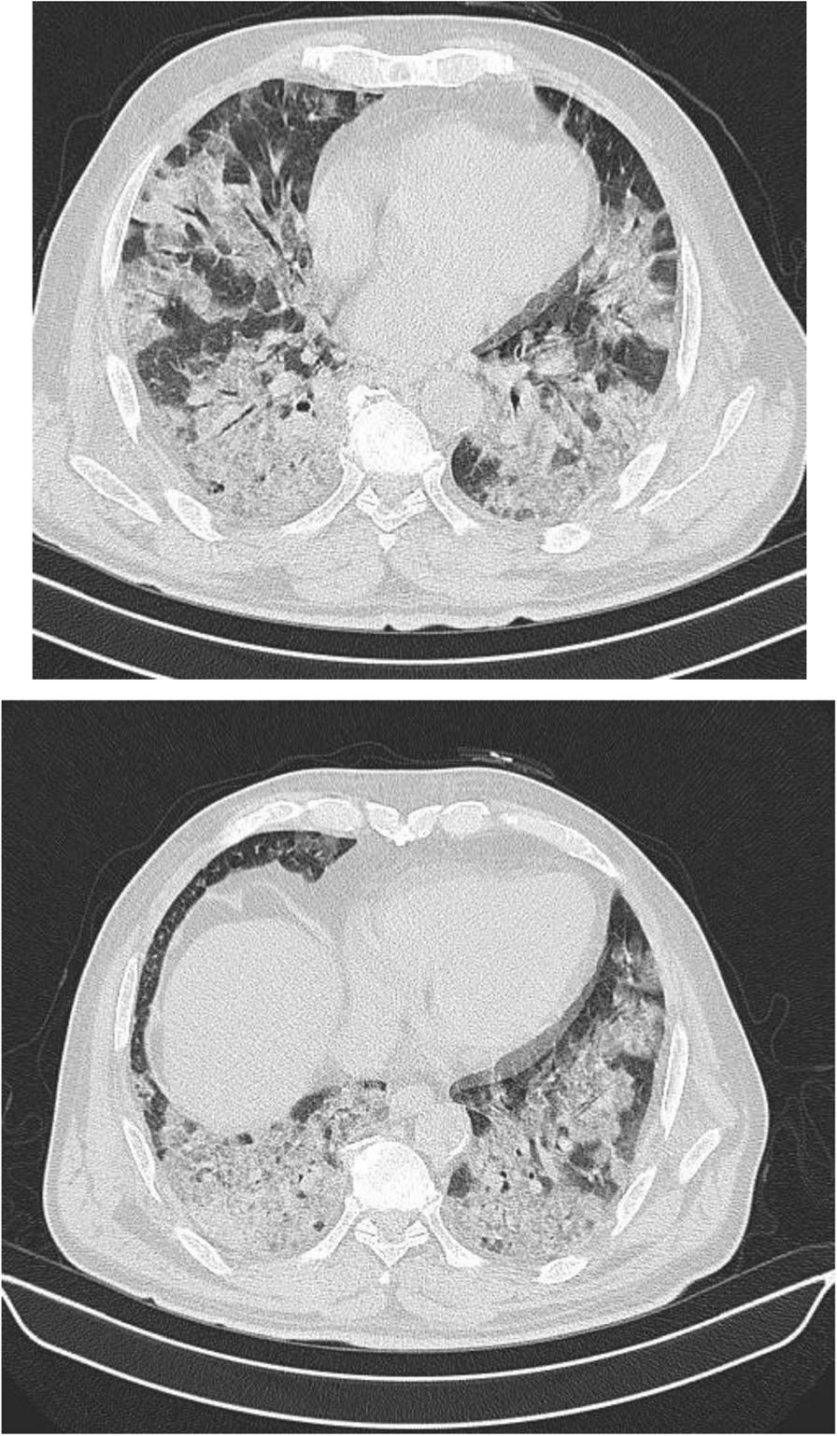
Patient with COVID-19 pneumonia 10 days after onset of symptoms. Wide areas of ground glass appearance and consolidation with fibrous bands

**Fig. 5:**
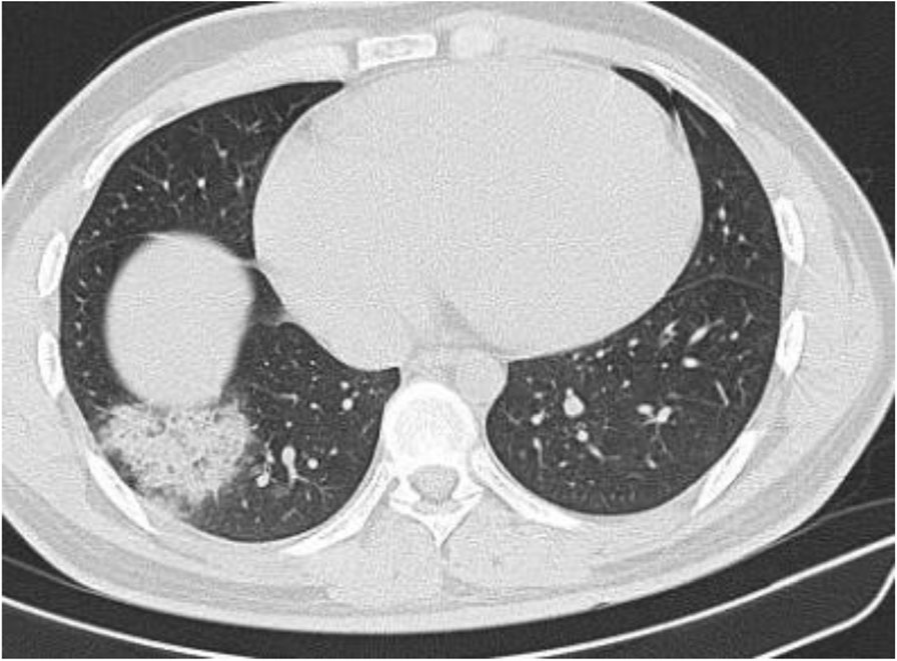
Patient about 24 days after onset of symptoms. A well-defined area of consolidation/fibrosis seen in the right lower lobe, no other abnormality was noted in both lungs

**Fig. 6:**
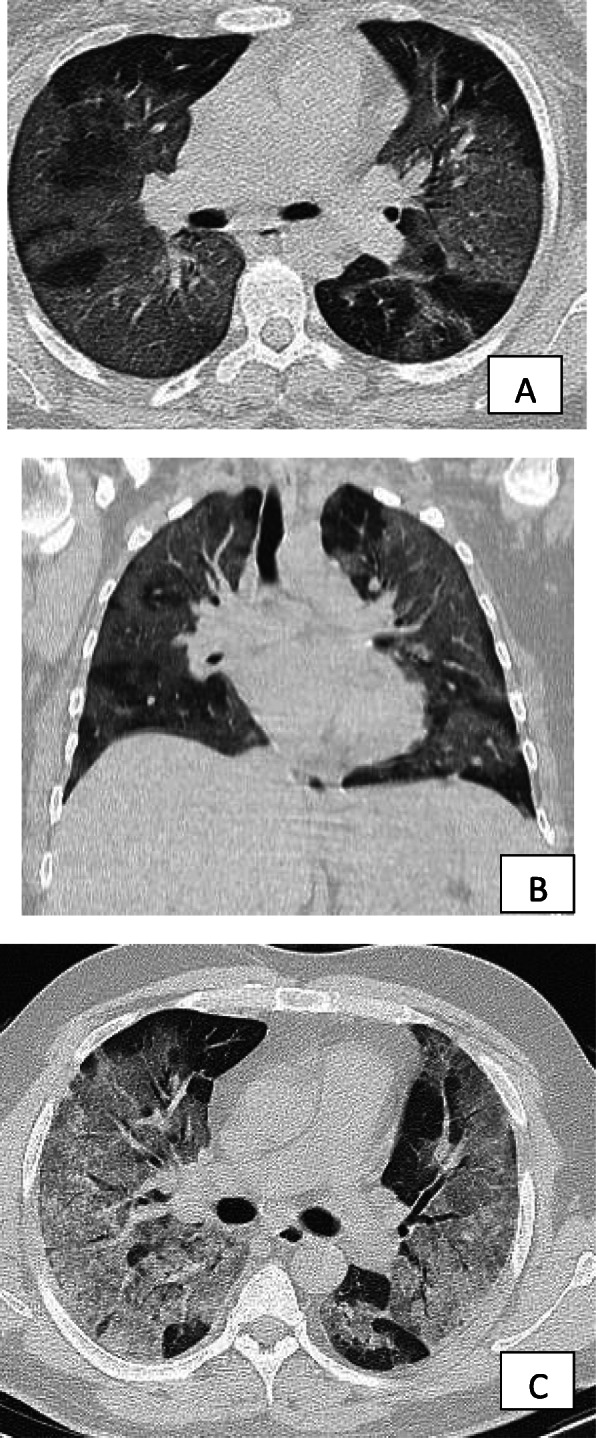
Two cases of white lung. **a**, **b** Patient with ground glass infiltration diffusely affecting both lungs giving the lung white out appearance. **c** Another case with relatively dense ground glass infiltration and consolidation diffusely affecting both lungs with air bronchogram

In patients with COVID-19 pneumonia, the most common findings in chest CT is GGO, which is usually described as patchy, peripheral, bilateral, and subpleural. Bao et al. [18] in a meta-analysis of 13 studies found that GGO was the most common manifestation, reported in 83.31% of cases. The meta-analysis involved 13 studies; GGO was the main finding in 11 of them. The two studies which did not report GGO were not radiological studies but were clinical studies, and they only reported bilateral abnormalities in the CT chest, and they should be excluded in our opinion [19, 20]. In another meta-analysis by Zhu et al. [21] involving 32 articles and 4121 patients, they reported ground glass opacification as the most common finding (68.1%). The relative low prevalence of GGO in this meta-analysis is because of the marked heterogeneity in the articles concerned mostly about clinical or laboratory findings. The ground glass opacification is the main CT chest findings in all articles published in radiology journal or other imaging journals [22–42]. For example, Guan et al. [37] in a study including 53 patients with COVID-19 reported GGO in all patients (100%). Ng et al. [15] reported 86% incidence of GGO, and the rest of the patients had GGO with consolidation. The expert recommendations from the Chinese Medical Association Radiology Branch classified the CT manifestations according to the appearance of GGO into four stages [43]; the early stage (Fig. 1) is characterized by dilatation of capillaries and engorgement of vessels, mild fluid exudates in the alveoli, and interstitial edema, resulting in single or multiple patchy ground glass opacities. The ground glass opacities are mostly peripheral and subpleural. The second stage is the advanced stage (Figs. 2 and 3) in which the lesions increase in density and size, forming mixed pattern of GGO and consolidation with or without air bronchogram. The cause of this appearance is the exudation into the alveolar space and the lung interstitium [10, 44]. The third severe stage in which there is fibrous exudates into the alveoli reflected in the chest CT as wide areas of consolidation with air bronchogram, with the non-consolidated area showing patchy ground glass infiltration (Fig. 4). In the 4th dissipation stage, the consolidation and ground glass infiltration gradually resolves, with small areas of residual fibrosis (Fig. 5). In some cases, the diffuse ground glass infiltration may give the lungs a white lung appearance (Fig. 6).

In summary, GGO is the most common and the earliest sign of COVID-19 pneumonia.

### Consolidation and air bronchogram

Consolidation is defined as an area of increased attenuation which obscures the bronchial and vascular markings and caused by filling the alveolar spaces by fluid, exudates, transudate, blood, or neoplastic cells [45]. Consolidation in COVID-19 pneumonia tends to be patchy or segmental, irregular or nodular, and mainly subpleural and peripheral with reported incidence 2–64% depending on the duration of the illness [46, 27, 47]. Consolidations usually appear after 10–12 days of the onset of symptoms, after the appearance of GGO. Yuan et al. [48] reported high mortality in patients with consolidation. Li et al. [46] in a series including 83 patients also reported consolidation in patients with severe or advanced disease. In a study by Song et al. [24], the incidence of consolidation was significantly higher in older patients (> 50 years) than younger patients and in patients with symptoms more than 4 days (Figs. 3, 4, and 5).

**Fig. 7:**
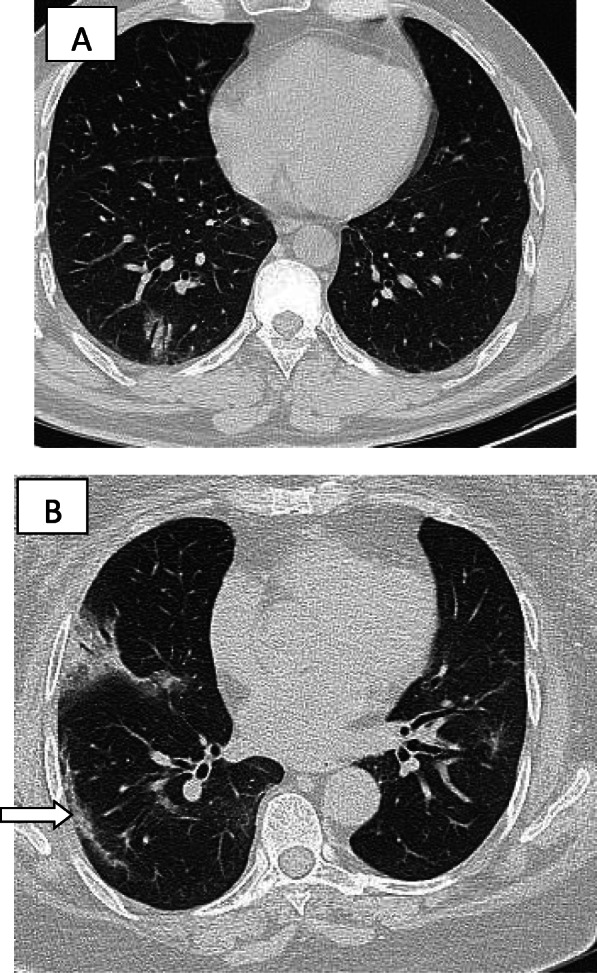
**a** Ground glass infiltration with air bronchogram. **b** Small area of consolidation with air bronchogram. Note the presence of sub-pleural line (white arrow)

Air bronchogram, which is defined as air-filled bronchi in area with high density, has variable incidence in different reports ranging from 28 to 80% of patients [24, 49]. Air bronchogram is usually a sign of advanced disease, usually seen after the second week from the onset of symptoms. Air bronchogram can be seen in both GGO and consolidation (Fig. 7).

### Reticulations

**Fig. 8:**
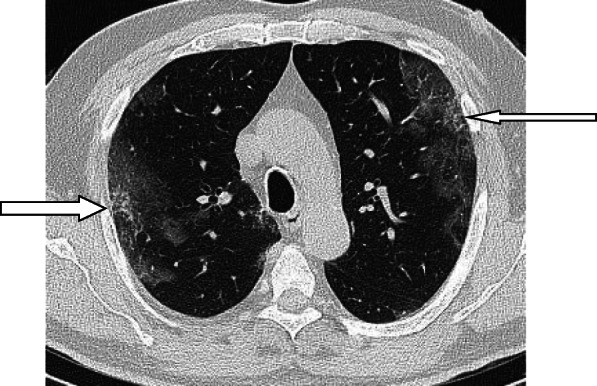
Patient with COVID-19 pneumonia, 8 days after appearance of symptoms with ground glass infiltration and reticulations

Reticulations which appear as lineal interlobular or intralobular density are a relatively late finding in patients with COVID-19, and its reported incidence is 48.5–59% [32, 22]. The appearance of reticulations is usually associated with clinical progression of the disease. The cause of reticulations is probably caused by lymphocyte infiltration of the interstitial tissues with interlobular and septal thickening. In some studies, the reticular pattern was a common pattern, considered the third common sign after GGO and consolidation [22] (Fig. 8).

### Crazy paving sign

**Fig. 9:**
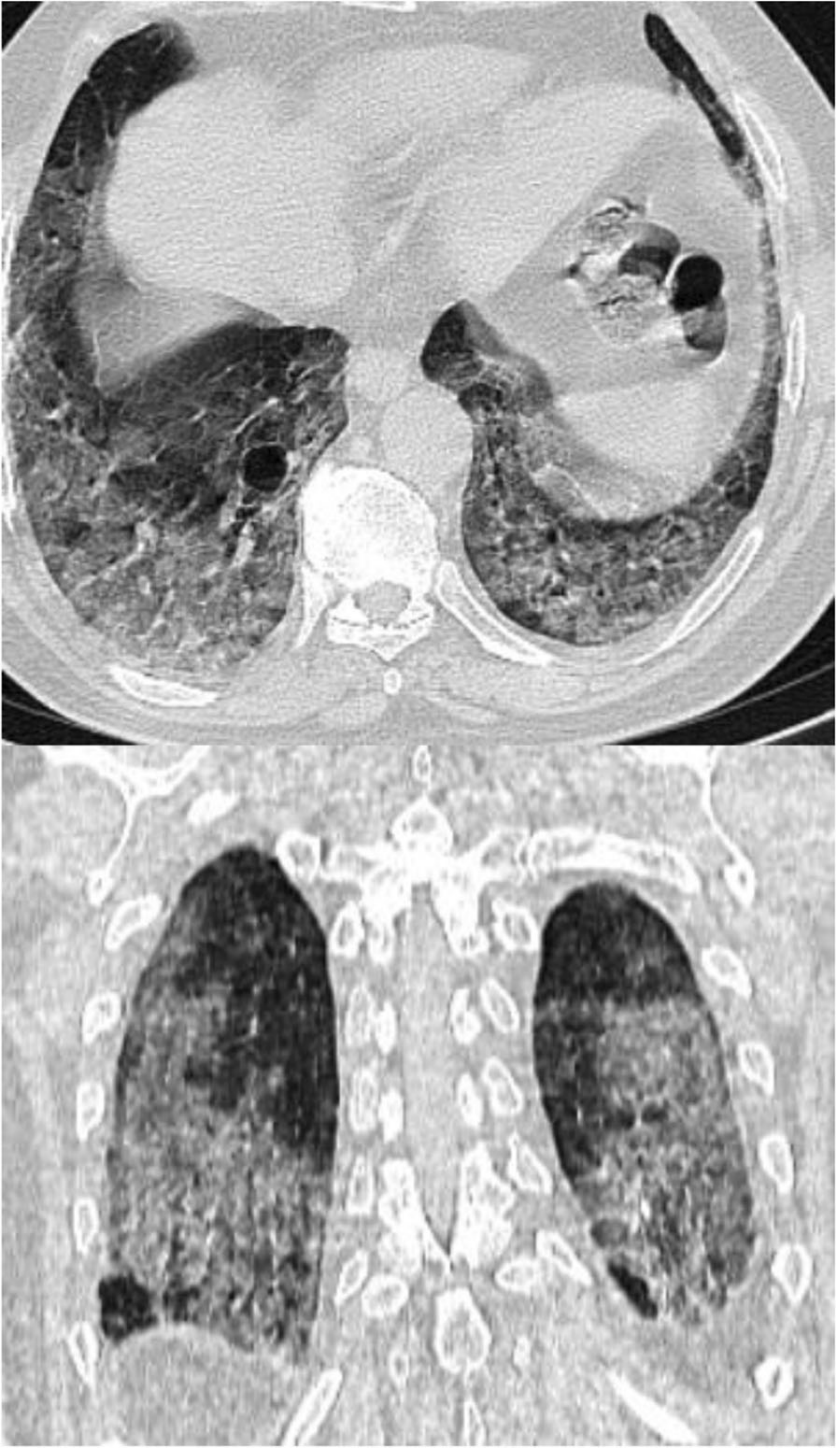
Axial and coronal CT images of patient with COVID-19, 10 days after the onset of symptoms, showing extensive ground glass infiltration with crazy paving infiltration. The appearance of crazy paving appearance indicates a progressive disease

**Fig. 10:**
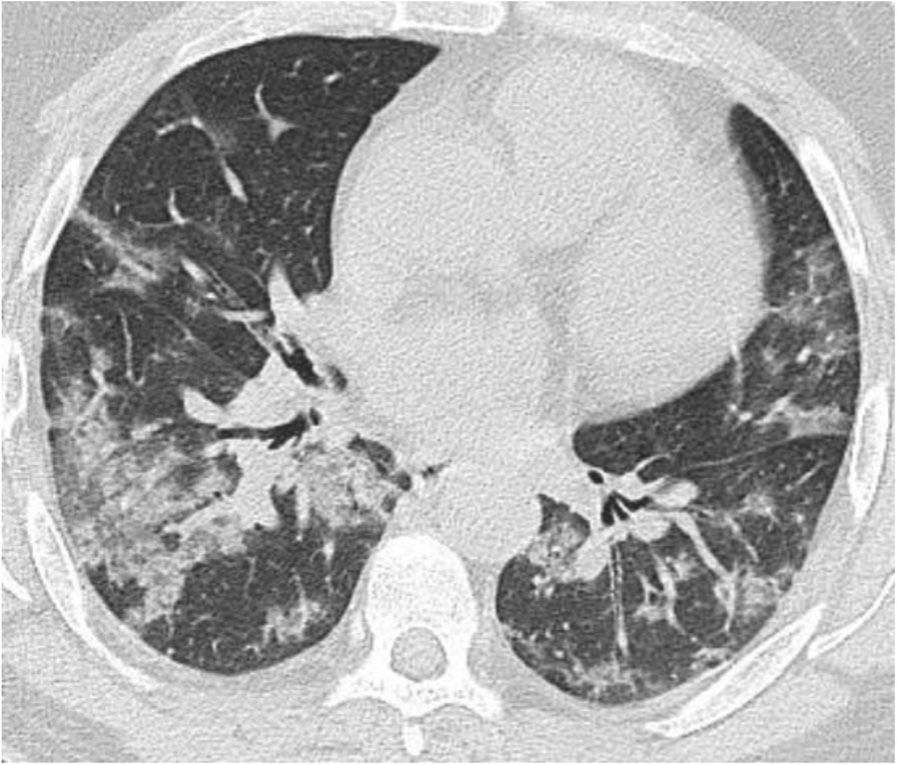
Patient with advanced COVID-19 and crazy paving appearance

The crazy paving signs represent thickened interlobular septa superimposed on GGO. This sign represents alveolar edema and interstitial inflammatory reaction [45, 47]. In the meta-analysis of Bao et al. [18], the crazy paving sign had incidence of 14.81% (95% CI 6.61–25.99%). On the other hand, some articles reported higher incidence like study performed by Guan et al. [37], who reported 89.4% incidence of crazy paving sign, and they thought this sign was due to hyperplasia of interlobular and intralobular interstitia. Interestingly, though the crazy paving sign is a sign of progressive disease and its appearance may indicate that the disease is entering the peak stage [47], yet it is the first CT sign to resolve in the absorptive stage while the consolidation, and GGO may persist for up to 26 days [50] (Figs. 9 and 10).

### Nodules

**Fig. 11:**
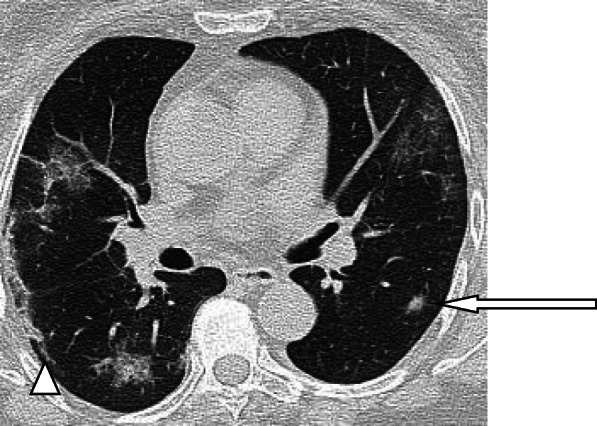
Patient with COVID-19 pneumonia, with left lower nodule (arrow), note also the presence of ground glass patches and subpleural line (arrow head)

**Fig. 12:**
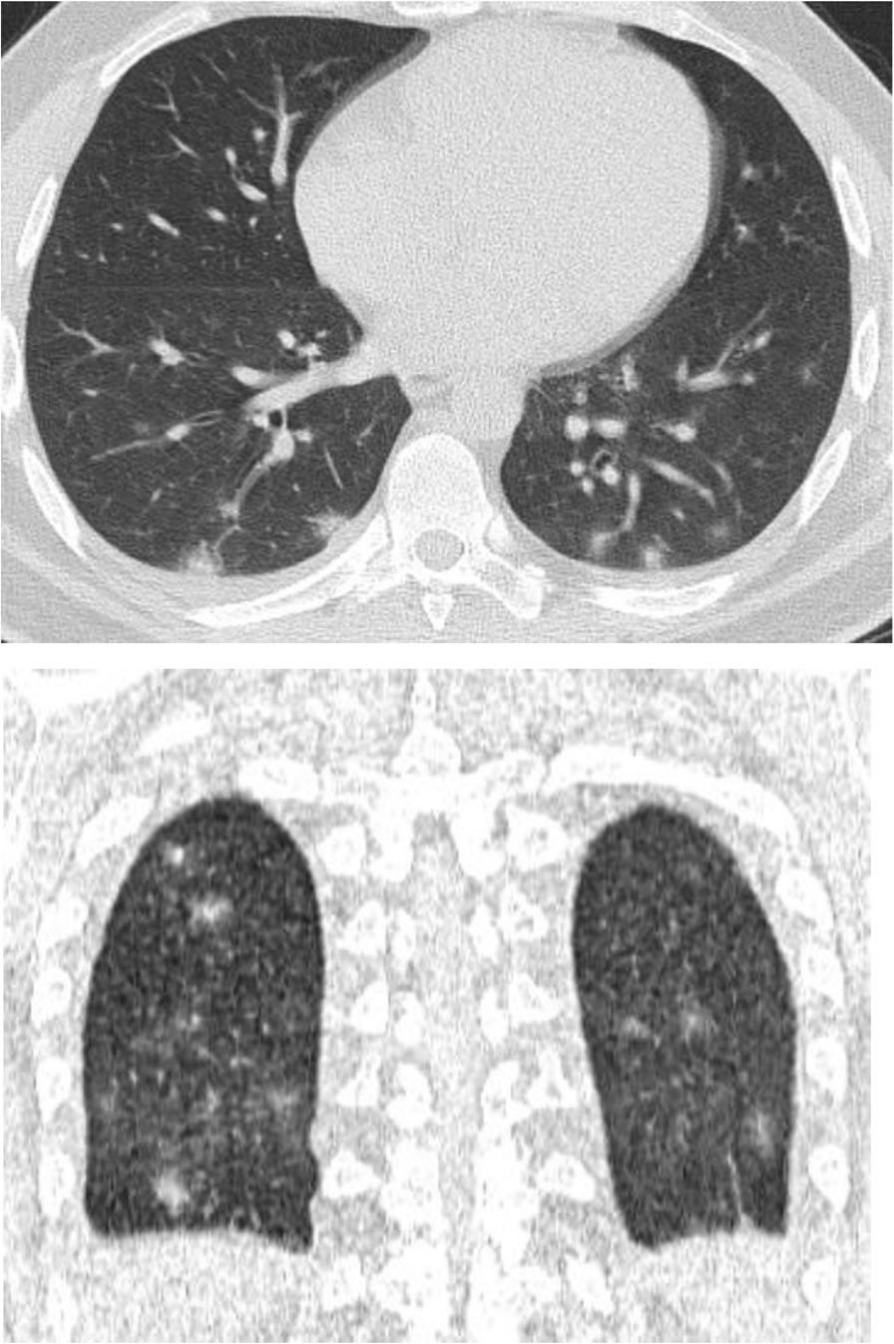
Patient with COVID-19. Multiple small nodules in both lungs, mainly subpleural and posterior

A nodule is an opacity less than 3 cm in diameter with regular or irregular outline. In general, viral pneumonitis is characterized by the presence of nodules [51]. The reported incidence of pulmonary nodules in patients with COVID-19 pneumonia is 3–13% [23] and may be associated with surrounding halo [52]. Yang et al. [53] considered the appearance of nodules in CT chest a sign of progressive course (Figs. 11 and 12).

### Subpleural curvilinear line (Fig. 13)

Subpleural curvilinear line appears as thin linear shadow 1–3 mm in thickness, parallel to and lying within 1 cm from the pleural surface. About 20% of patients with COVID-19 have these signs, and it may represent edema or fibrosis [32, 54]. Zhou et al. [55] reported this sign in 9.7% of their studied patients. Rouhezamin et al. [56] considered the presence of this sign as a differentiating sign of COVID-19 from lung contusion. In another study which included 62 patients, the subpleural line was reported in 21 (33.9%) patients, and it was more common in advanced disease than early disease [57].

**Fig. 13:**
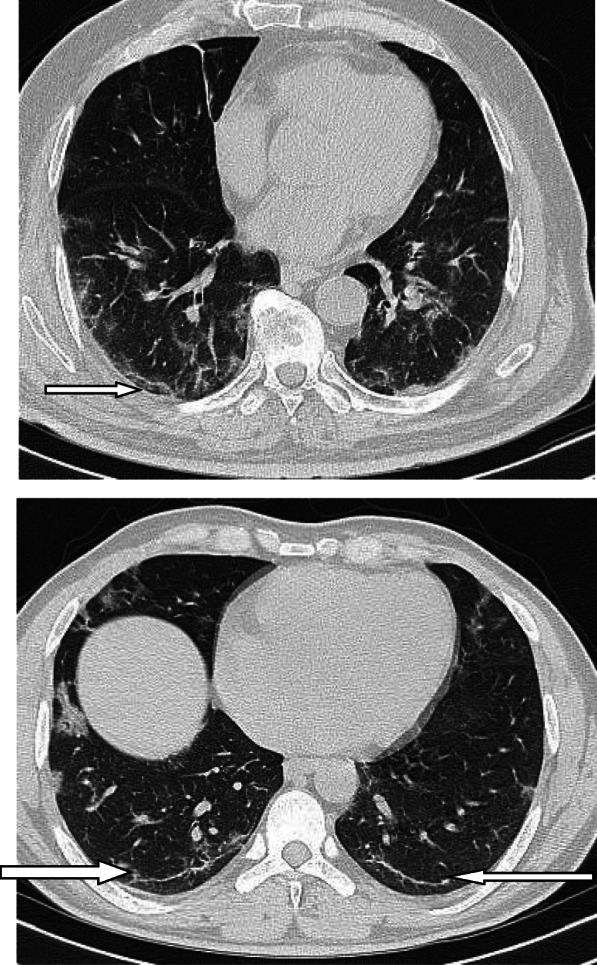
Two different patients with COVID-19 pneumonia with subpleural lines (arrow), note also the presence of patches of ground glass infiltration

### Halo sign (Figs. 14, 15, and 16)

Halo sign is defined as ground glass opacity surrounding a nodule or mass. Previously, this sign is considered a manifestation of fungus infection, viral pneumonia, or hypervascular metastasis [52]. In a recent study by Bai et al. [58], they reported halo sign in 26% of patients with COVID-19 pneumonia and 21% of cases with other viral pneumonia, and they found it a non-helpful sign in differentiating COVID-19 pneumonia from other viral pneumonia.

**Fig. 14:**
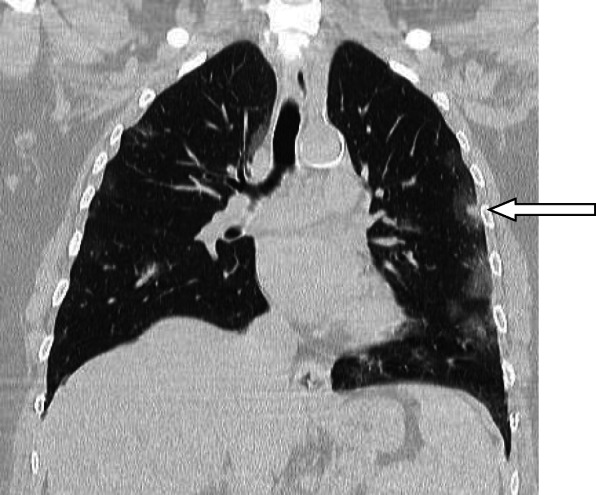
Patient with COVID-19 pneumonia with multiple peripheral areas of GGO. Halo sign is seen in the left lung

**Fig. 15:**
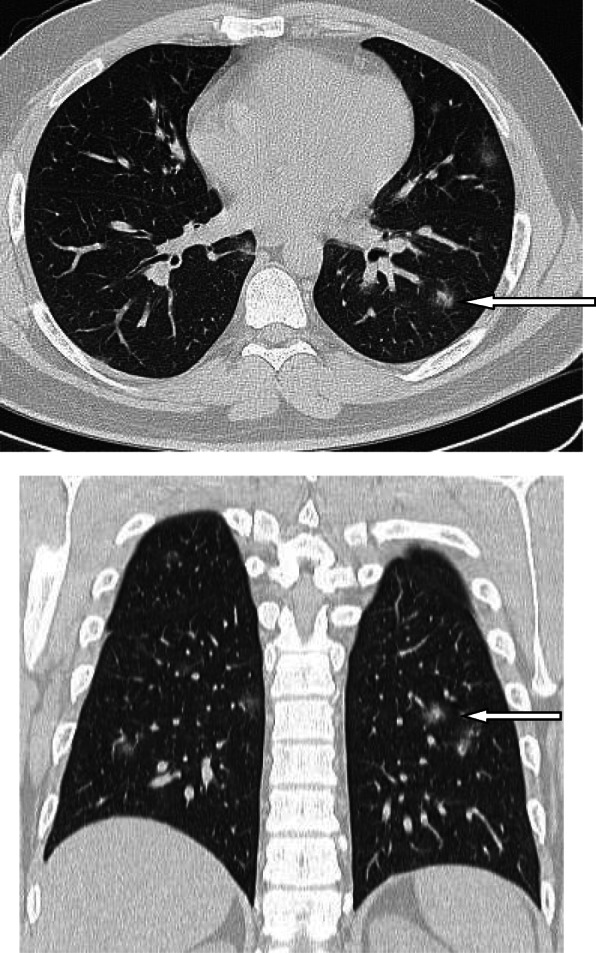
Patient with COVID-19, with few patches of GGO, with halo sign in few lesions

**Fig. 16:**
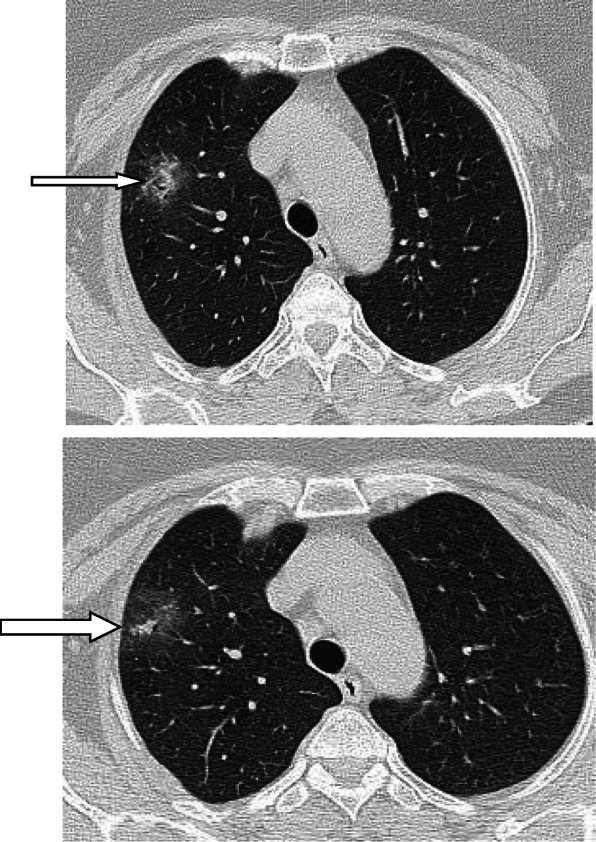
Another patient with patches of ground glass infiltration and halo sign

### Inverted (reversed) halo sign or atoll sign

**Fig. 17:**
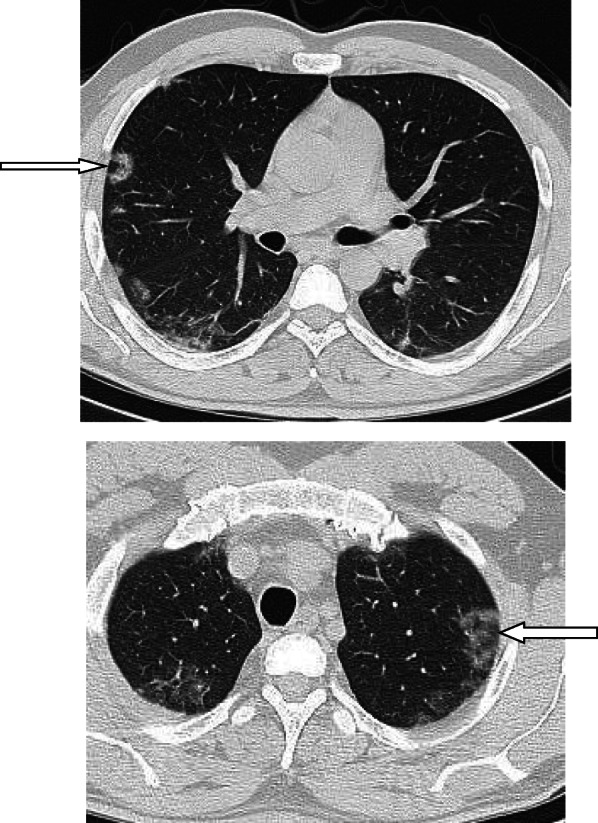
Patient with COVID-19 pneumonia with reversed halo sign

The reversed halo sign represents an area of GGO surrounded by near complete ring of consolidation [45]. The proposed mechanisms in COVID-19 pneumonia is either disease progression with development of consolidation around area of GGO or consolidated area with resolution of the central area leaving area of decreased density [7, 49]. The reversed halo sign is usually seen in relatively long time onset of symptoms, and the presence of this sign suggests that organizing pneumonia may be one of the mechanisms of lung injury in COVID-19 pneumonia [59, 60] (Fig. 17).

### Subpleural transparent line

**Fig. 18:**
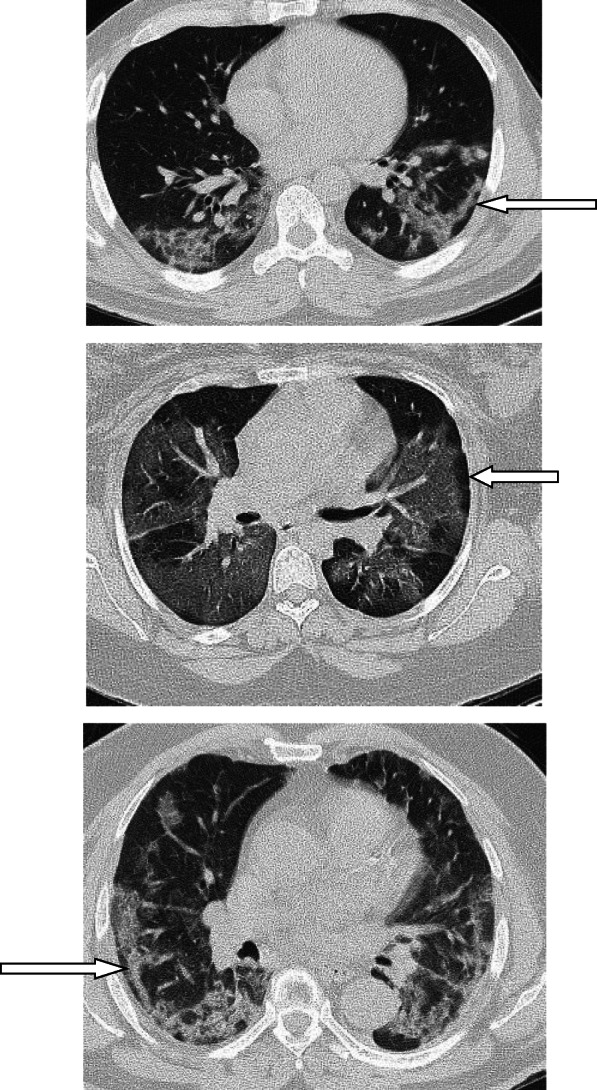
Subpleural transparent line in three different patients with COVID-19 pneumonia

Subpleural transparent line is defined as thin and transparent line between the areas GGO or consolidation and the visceral pleura, and it was reported in 53.2% in one study [57]. Another study by Zhou et al. [61] involving 100 patients reported the incidence of transparent line to be 45.3% in the early stage, 47.7% in the advanced stage, and 6.5% in the absorption stage, and they suggested that the presence of this sign indicates advanced stage (Fig. 18).

### Air bubble sign (vacuolar sign)

**Fig. 19:**
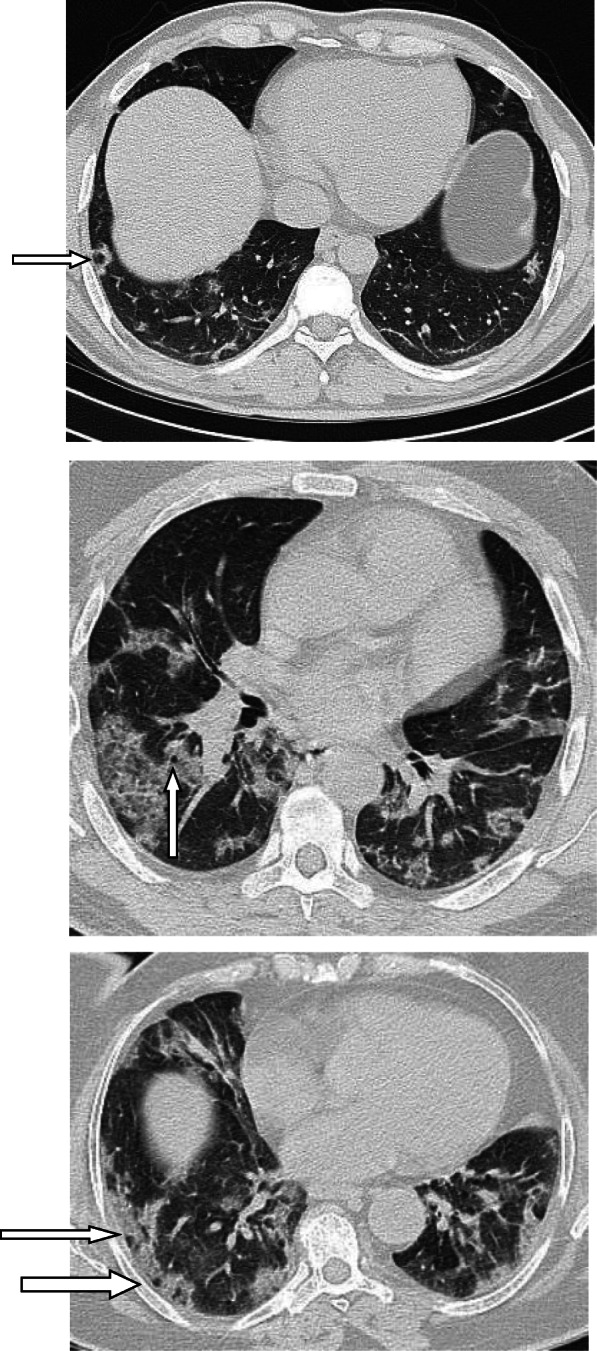
Air bubble sign (vacuolar sign) in three different patients (arrows)

Air bubble sign (vacuolar sign) refers to a small air-containing space < 5 mm in length within the lung lesion [57]. Some authors called it small cystic changes [22] and cavity sign [62]. Generally, Zhou et al. [57] reported it in 54.4% of their patients, and they considered it as a sign of progressive disease (Fig. 19). The air bubble sign may be due to dilatation of physiological spaces or transverse section of a bronchus within an area of consolidation. It may represent an early sign of consolidation resorption.

### Vascular enlargement

Vascular dilatation within or around the lesions in CT chest is a common finding in patients with COVID-19. In a study which included 51 patients, vascular enlargement was reported in 42 (82.4%) patients [63]. This signs were also observed in the studies of Lomoro et al. [64] (23.8%), Zhao et al. [65] (71.3%), and Zhou et al. [57] (45.2%), and it has been correlated to hyperemia induced by acute inflammatory response and the disruption of the capillary wall inflammatory mediators [54].

**Fig. 20:**
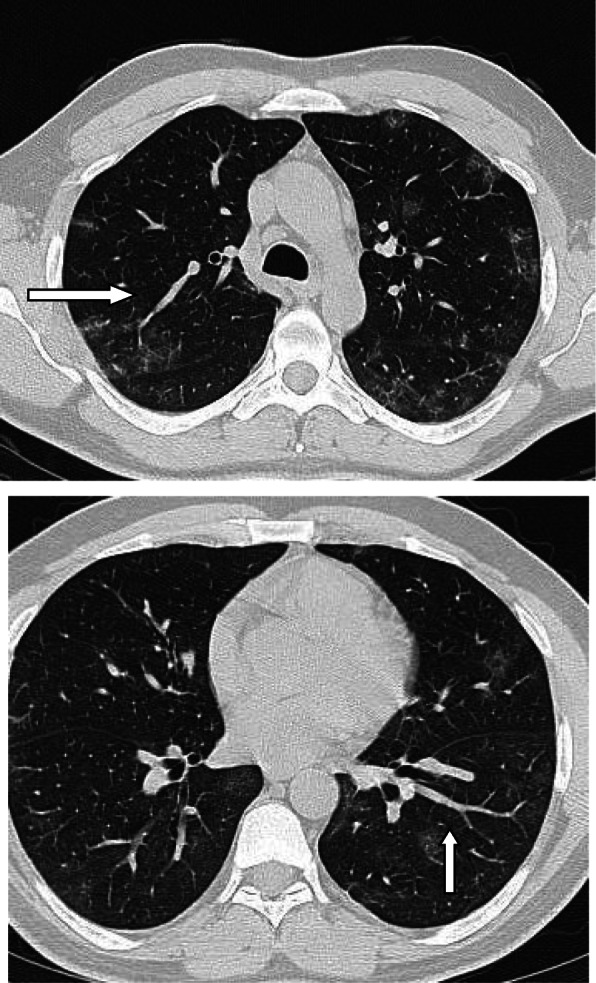
Dilated pulmonary vessels in patient with acute COVID-19 pneumonia

Interestingly, the vascular enlargement was reported in asymptomatic patients with COVID-19, as an association with GGO [66]. In a study by Dai et al. [40], they reported vascular enlargement associated with GGO as the most common findings in patients with COVID-19, and they attributed it to vascular congestion and dilatation of small vessels. The vascular enlargement associated with GGO can be considered as early predictor of lung impairment (Fig. 20).

### Bronchial changes

Bronchial wall thickening in patients with COVID-19 pneumonia has been reported in 20% of patients, and it is attributed to inflammatory changes in the bronchial wall, bronchial obstruction, and fibrosis [67, 32]. In a study including 83 patients, bronchial wall thickening was found in patients with severe or progressive disease [67]. Bronchial wall thickening is more common in pediatric patients than adult patients [68].

**Fig. 21:**
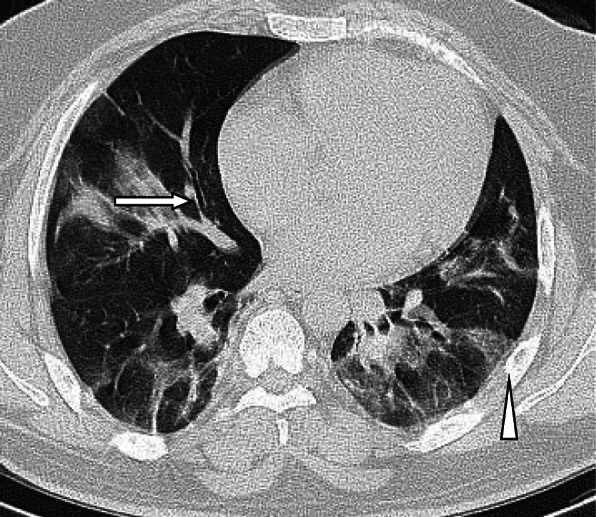
Mild bronchial dilatation, bronchial wall thickening (arrow). Note the presence of spider web sign in the left lower lobe (arrow head)

Bronchiectasis was reported in some cases of COVID-19 patients [69]. Zhao et al. [65] reported bronchiectasis in 52.5% of their patients, and they considered this sign, together with architectural distortion and pleural effusion, a reflection of the severity of the disease and expression of viral load and virulence of the disease (Fig. 21).

### Spider web sign (Fig. 21)

Originally described by Wu et al. [32], spider web sign represents subpleural triangular area of GGO, with web-like thickening of the interlobular septa and retraction of the adjacent pleura. In the meta-analysis study by Zhu et al. [21], including 4121 patients, spider web sign was reported in 39.5% of patients in eleven studies. They considered it a common sign of COVID-19 pneumonia.

### Pleural changes

**Fig. 22:**
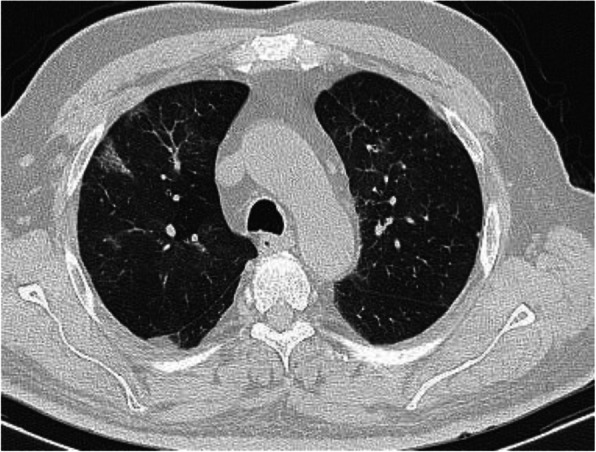
Pleural thickening in the right side in patient with COVID-19 pneumonia

Pleural thickening and pleural effusion are relatively less common findings in patients with COVID-19. The reported incidence of pleural thickening is about 27–32% [21, 27]. The incidence of pleural effusion is less common (2–5%). There is agreement between studies that the presence of pleural effusion carries a poor prognosis and reflects high viral load and high virulence [65, 67] (Fig. 22).

### Pericardial effusion

Pericardial effusion is relatively uncommon in patients with COVID-19. In the study by Li et al. [63], they divided their patients into two groups, group with severe clinical symptoms and ordinary group, and they found pericardial effusion in 5% of patients with severe disease and not reported in the ordinary group; they concluded that pericardial effusion is an indication of severe disease.

### Mediastinal lymphadenopathy (Fig. 23)

Mediastinal lymph nodes are said to be enlarged when the short axis diameter is 1 cm or more [45]. In patients with COVID-19, mediastinal lymphadenopathy is not a typical feature, with incidence of 1–6% [22, 65, 67]. In general, the presence of enlarged lymph nodes is considered a sign of severe or critical disease [67]. Also, the presence of enlarged lymph nodes may indicate superimposed bacterial infection [70]. Recently, Valette et al. [71] reported high incidence of lymphadenopathy (66%) in patients admitted to ICU with severe respiratory distress syndrome, with some lymph nodes large in size, and they considered it a sign of critically ill patients.

**Fig. 23:**
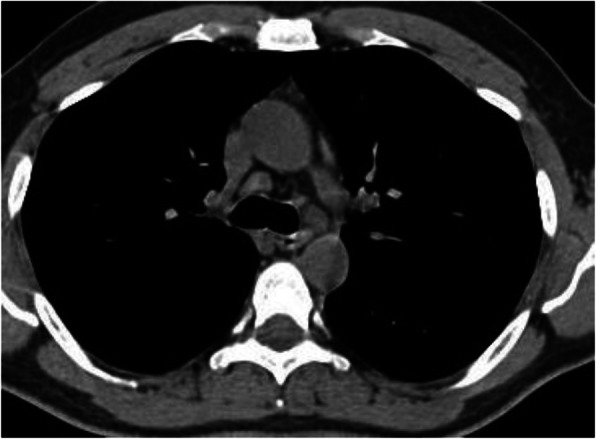
Enlarged mediastinal lymph nodes in patient with COVID-19

### Fibrosis

Lung fibrosis and fibrous strips have been reported in patients with COVID-19, with reported incidence about 17% [23]. Some authors consider it a sign of regression of disease severity and carries good prognosis [23], but other authors consider it a sign of severe disease [72] or a warning sign of development of interstitial fibrosis [73].

### Anatomical lesion distribution

Bao et al. [18] in a meta-analysis involved 13 studies; the disease was bilateral in 81.8% of patients. The lesions are more common in the peripheral areas (76.95%). Few lesions were located in the central (peribronchovascular) area. The lower lobes are more commonly involved; the right lower lobe and left lower lobe were the most commonly involved, 87.21% and 81.41% respectively, and both lower lobes in 65.22%. The upper lobes were involved in 65.22% and 69.43% for the right and left sides respectively. There are about 39.54% of patients with all lobes affected and 20.51% patients with four lobes affected.

In general, the disease most commonly affects both lungs, the lower zones more commonly affected and the right middle lobe is the least involved one. Also, patchy multifocal distribution is more frequent compared with diffuse disease, but unilateral or unifocal affection can occur. The peribronchial distribution is rare and considered atypical [24, 74].

### Normal CT chest in patients with COVID-19 pneumonia

CT plays a very important role in the diagnosis and management of COVID-19 pneumonia. However, multiple studies reported normal CT chest in patients with COVID-19 pneumonia. Bernheim et al. [27] reported that 20 out of 36 patients (56%) had normal CT chest within 2 days from onset of symptoms. Ai et al. [28] reported 21 of 601 (3%) patients with positive RT-PCR had normal CT chest. Chung et al. [34] reported three of 21 patients with positive coronavirus and normal CT chest, with one of these patients progressed 3 days later and developed a solitary rounded ground-glass lesion. They thought that chest CT lacks complete sensitivity and cannot alone exclude the disease, particularly early in the infection. Also, this may be related to the incubation period of the disease or there may be a prodromal phase where viral infection symptoms appear before the appearance of imaging manifestations. The Centers for Disease Control and Prevention has noted that symptoms of COVID-19 pneumonia may appear in as few as 2 days or as long as 2 weeks after exposure, which is similar to the incubation period of MERS [34].

#### CT score

The total severity score was presumed by Chung et al. [34] based on assessment of the degree of involvement of each of the five lung lobes by ground glass infiltration or other abnormality and classification as none (0%); minimal (1–25%), corresponded to a lobe score 1; mild (26–50%), corresponded to a lobe score 2; moderate (51–75%), corresponded to lobe score 3; or severe (76–100%), corresponded to lobe score 4. An overall lung “total severity score” was achieved by summing the five lobe scores, with range of possible scores 0–20.

Later, Zhang et al. [75] in a study which included 84 patients found the severity score to be correlated with the laboratory data in the early and progressive phases, and the severity score may be a useful tool in patient assessment. However, the severity score did not reduce and lag behind the laboratory findings in the late stage.

Another scoring system was suggested by Yuan et al. [48] who graded the CT attenuation into three grades: 1 for normal attenuation, 2 for ground glass opacification, and 3 for consolidation. The degree of lung involvement was evaluated for 6 lung regions: upper, middle, and lower lung on each side. The involvement of each lobe was graded using a 5-point scale: 0, no involvement; 1, less than 25%, 2, 25–50%; 3, 50–75%, and 4, > 75%. The highest CT score was 72. They used a cutoff value of 24.5 and expected mortality with a sensitivity of 85.6% and a specificity of 84.5%, and they concluded that the high CT score correlates with the patient’s mortality.

In our opinion, the CT score by Yuan et al. [48] is more applicable and put into consideration the density of the lesion and not only the width of involvement.

#### Accuracy of CT chest in diagnosis of COVID-19 pneumonia

The reported sensitivity of CT in the diagnosis of COVID-19 is 60–98%, and the reported specificity is 25–56% [28, 35, 76, 77]. The reported positive and negative predictive values are 92% and 42% respectively [76]. The low specificity and negative predictive values suggest that CT is unsuitable as a screening tool [74].

A meta-analysis performed by Xu et al. [78], involving 16 studies and 3186 patients, emphasized the high sensitivity (92%) and low specificity (25–33%) of CT in the diagnosis of COVID-19. The high sensitivity of CT led to adoption of chest CT as a diagnostic criterion in the fifth edition of the Diagnosis and Treatment Program of 2019 New Coronavirus Pneumonia proposed by the National Health Commission of China [79]; however, CT chest was later removed in the sixth edition [80]. Generally speaking, in the epidemic areas, the addition of CT as diagnostic criterion will allow early diagnosis and effective control of the epidemic.

The low specificity of CT chest in the diagnosis of COVID may be due to negative CT in early disease or mild disease. Also, it may be because of overlap between COVID pneumonia and other viral pneumonia. In the current pandemic situation, the sensitivity is more important than specificity because there is need to isolate any suspicious case to prevent cross infection [78]. It is to be mentioned that CT alone cannot diagnose COVID-19. According to the guideline of Diagnosis and Treatment of Pneumonitis Caused by 2019-nCoV (trial sixth version), the chest CT findings should be a diagnostic criterion only when combined with epidemiology history, clinical manifestations, and laboratory results [80].

#### Differential diagnosis

The pneumonia from bacterial origin is easily differentiated from COVID-19 pneumonia because it is characterized by airspace consolidation with lobar or segmental distribution with additional ground glass attenuation, centrilobular nodules, bronchial wall thickening, and mucoid impactions which is quite different from COVID-19 pneumonia presentation [81].

The diffuse GGO in *Pneumocystis jiroveci* pneumonia in immunocompromised persons can be differentiated from COVID-19 pneumonia because it spares the subpleural spaces [82].

The main challenge is to differentiate COVID-19 pneumonia from pneumonia due to other viral causes with overlapping CT features. The abnormalities in COVID-19 pneumonia tend to be peripheral lesions and usually not associated with pleural effusion or lymphadenopathy, yet, in the context of COVID-19 pandemic, we should always suggest COVID-19 as the cause of GGO in patients with fever and respiratory complaint. In a recent study which included 122 patients, the authors found that the rounded opacities associated with interlobular septal thickening, in the absence of nodules and tree-in-bud sign in a lung periphery, favors the diagnosis COVID-19 [83].

#### Artificial intelligence (AI)

A major recent advance in the current decade is the artificial intelligence (AI). The application of diagnostic AI models would enable prioritization and help reduce reporting time. The AI would be extremely useful in the epidemic situation compensating the shortage of manpower and hospital beds. AI models for the chest radiographs and CT scans may help alleviate the work overload of radiologists and clinicians and enhance rapid diagnosis and management. Li et al. [84] found the deep learning model accurate with high sensitivity and specificity in the diagnosis of COVID-19 and differentiating it from other forms of pneumonia.

#### Chest CT compared to RT-PCR

The sensitivity of CT depends on the duration of symptoms. Bernheim et al. [27] reported negative CT in 56% of patients scanned in the first 2 days of symptom onset, whereas negative scans were demonstrated in 9% of patients scanned within 3–5 days, and 4% in patients scanned 6–12 days of symptoms, respectively.

Generally, CT appears to have a higher sensitivity than rRT-PCR test. Long et al. [85] reported higher CT sensitivity of 97.2% compared to initial rRT-PCR test sensitivity of 83.3%. The microbiological tests such as real-time polymerase chain reaction (RT-PCR) may not be available in an emergency setting, and their results take 4–5 days. On the other hand, computed tomography (CT) can be used as an important complement to RT-PCR for diagnosing COVID-19 pneumonia in the current epidemic context. Also, false negative result is obtained on RT-PCR when the viral load is insufficient [24].

In the study by Ai et al. [28] comparing the performance of RT-PCR and CT chest in 1014 patents, they found that 59% (601/1014) had positive RT-PCR compared to 88% (888/1014) positive CT chest. They found 60% of patients had positive CT findings before or parallel to positive RT-PCR, and nearly all patients (56/57) had positive CT before or within 6 days of positive RT-PCR. Moreover, 70% of patients with negative RT-PCR had typical appearance on chest CT. The RT-PCR can be also affected by the sampling technique, source of specimen (upper or lower respiratory tract), viral load, time of sampling, and the type of kit.

## Conclusion

CT plays a pivotal role in the diagnosis and management of COVID-19 pneumonia. The typical appearance of COVID-19 pneumonia is bilateral patchy areas of ground glass infiltration, more in the lower lobes. The appearance of other signs like consolidation, air bronchogram, crazy pavement appearance, and air bubble signs appear during the course of the disease. The appearance of pleural effusion or pericardial effusion carries a poor prognostic value. Thin section chest CT has a good sensitivity but low specificity in the diagnosis of COVID-19 pneumonia. Though RT-PCR is still the recommended screening test for COVID-19 pneumonia, we think that during the context of pandemic like the current situation, the CT chest can be used as a screening tool in symptomatic patients as it is cheaper, available, and time saving.

## Data Availability

Data are available upon reasonable request.

## Abbreviations

SARS: Severe acute respiratory distress syndrome
MERS: Middle East respiratory syndrome
RT-PCR: Reverse transcription polymerase chain reaction
GGO: Ground glass opacity
WHO: World Health Organization
NPV: Negative predictive value
PPV: Positive predictive value
